# Retrospective analysis of molecular scores for the prediction of distant recurrence according to baseline risk factors

**DOI:** 10.1007/s10549-016-3868-y

**Published:** 2016-07-22

**Authors:** Ivana Sestak, Mitch Dowsett, Sean Ferree, Frederick L. Baehner, Jack Cuzick

**Affiliations:** 1 Centre for Cancer Prevention, Wolfson Institute of Preventive Medicine, Queen Mary University, Charterhouse Square, London, EC1M 6BQ UK; 2 Academic Department of Biochemistry, Breakthrough Breast Cancer Centre, Royal Marsden Hospital, London, UK; 3 NanoString Technologies, Seattle, USA; 4 Genomic Health Inc., Redwood City, USA

**Keywords:** Molecular scores, Prognostic information, Differential effect, Age, Body mass index

## Abstract

Clinical variables and several gene signature profiles have been investigated for the prediction of (distant) recurrence in several trials. These molecular markers are significantly correlated with overall and late distant recurrences. Here, we retrospectively explore whether age and body mass index (BMI) affect the prediction of these molecular scores for distant recurrence in postmenopausal women with hormone receptor-positive breast cancer in the transATAC trial. 940 postmenopausal women for whom the Clinical Treatment Score (CTS), immunohistochemical markers (IHC4), Oncotype Recurrence Score (RS), and the Prosigna Risk of Recurrence Score (ROR) were available were included in this retrospective analysis. Conventional BMI groups were used (*N* = 865), and age was split into equal tertiles (*N* = 940). Cox proportional hazard models were used to determine the effect of a molecular score for the prediction of distant recurrence according to BMI and age groups. In both the univariate and bivariate analyses, the effect size of the IHC4 and RS was strongest in women aged 59.8 years or younger. Trends tests for age were significant for the IHC4 and RS, but not for the CTS and ROR, for which most prognostic information was added in women aged 60 years or older. The CTS and ROR scores added significant prognostic information in all three BMI groups. In both the univariate and bivariate analyses, the IHC4 provided the most prognostic information in women with a BMI lower than 25 kg/m^2^, whereas the RS did not add prognostic information for distant recurrence in women with a BMI of 30 kg/m^2^ or above. Molecular scores are increasingly used in women with breast cancer to assess recurrence risk. We have shown that the effect size of the molecular scores is significantly different across age groups, but not across BMI groups. The results from this retrospective analysis may be incorporated in the identification of women who may benefit most from the use of these molecular scores, but our findings need further evaluation before these scores can be used in clinical decision making.

## Introduction

Breast cancer is the most common cancer in women, and its incidence has increased over the past few years. Most women will be diagnosed with an oestrogen receptor (ER)-positive tumour, for which endocrine therapy will improve their outcome substantially [1]. The risk of a recurrence is specifically high for women with ER-negative breast cancer in the first 5 years after diagnosis. In contrast, women with ER-positive breast cancer remain at risk for recurrence even after 5 years of endocrine therapy, with an estimated annual excess rate of 2 % for at least 15 years.

In recent years, the development and use of multi-gene signatures for the identification of women at high risk of recurrence have increased noticeably. The 21-gene *Oncotype* Dx recurrence score (RS) [2] has been developed to classify women with early breast cancer into risk categories for recurrence and has been validated in several cohorts [3]. The RS improved risk stratification in postmenopausal patients in the transATAC (Arimidex, Tamoxifen Alone or Combined) trial [3]. Furthermore, the prognostic precision of RS was enhanced by incorporating classical clinicopathological parameters, clinical treatment score (CTS) [4, 5]. In the same transATAC trial, similar prognostic information was derived from four immunohistochemically measured markers (ER, progesterone receptor (PgR), Ki67 and HER2) integrated into the immunohistochemical markers (IHC4) score [4]. The Prosigna assay, based on the PAM50 gene signature, was developed to determine the intrinsic subtype of a tumour and a Risk of Recurrence Score (ROR) that is correlated with the probability of distant recurrence [6, 7]. The Prosigna ROR score was shown to add significant prognostic information over standard clinicopathological variables in the transATAC trial [8] and the ABCSG-8 trial [9]. In a recent publication [10], a combined analysis of these two trials showed that the ROR predicted late distant recurrence beyond that of clinical parameters.

Other molecular signatures, such as the EndoPredict [11], Breast Cancer Index [12], Mammaprint [13, 14], have also been developed for the identification of breast cancer patients who are at high risk of a recurrence. However, all the above signatures have in common that apart from clinicopathological features, no other non-clinical risk factors have been taken into account when their prognostic ability was developed and investigated. It is well known that age, body mass index (BMI), previous hormone replacement therapy (HRT) are the risk factors for the development of breast cancer [15–19]. It is therefore important to assess the value of incorporation of these parameters when analysing the prognostic ability of multi-gene signatures for the prediction of recurrence. The transATAC study offers a great opportunity to analyse the impact of baseline risk factors on the prediction of recurrence, as there is a median of 10 years follow-up on all patients, and data on the prognostic relevance of four clinical/multi-gene signatures are available.

## Methods

The main Anastrozole Tamoxifen Alone or in Combination (ATAC) trial evaluated the efficacy and safety of anastrozole, tamoxifen, or the combination in postmenopausal women with localised breast cancer [20]. For the transATAC protocol, formalin-fixed paraffin-embedded blocks from primary tumours were collected [21]. For this retrospective analysis, 940 women (84.0 %) from the transATAC study with hormone receptor-positive breast cancer who did not receive chemotherapy, randomised to either tamoxifen or anastrozole, and for whom we had data on all four scores available, were included. The IHC4 and CTS were developed on the transATAC dataset and have been described in detail previously [4]. In brief, the CTS contain information on nodal status, grade, tumour size, age, and treatment received. The IHC4 score was used as calculated previously [4]. The 21-gene-based *Oncotype* Dx RS was developed in women with hormone receptor-positive, node-negative breast cancer treated with tamoxifen [2]. The signature is based on 16 breast cancer-specific genes and five reference genes, including information on proliferation, oestrogen-related genes, invasion, HER2, and other factors [2]. The Prosigna ROR score is based on a 50-gene test [6, 7] and is derived from an expression profile of the 50 genes analysed on the NanoString nCounter Dx analysis system and also includes information on tumour size. A 46-gene subset of the PAM50 genes plus tumour size was used to calculate a predefined ROR score [22].

The primary objective of this study was to determine if non-clinical baseline factors affect the prognostic performance of clinical and multi-gene signatures for the prediction of distant recurrence in the transATAC study. Baseline (risk) factors included in this analysis were age and BMI (conventional groups: <25 kg/m^2^, 25–30 kg/m^2^, >30 kg/m^2^), previous HRT use, smoking status, hysterectomy, treatment with radiotherapy, and surgery type (mastectomy vs. breast conserving surgery). The time from randomisation to first distant recurrence was the prospectively defined primary endpoint. Death before distant recurrence was treated as a censoring event. The association between clinical/multi-gene scores, baseline risk factors, and distant recurrence was assessed using hazard ratios derived from Cox proportional hazard models with associated 95 % confidence intervals (CI). For multivariate analyses, each multi-gene signature was added separately to CTS to determine the prognostic information added by that score within a baseline risk group. All Hazard ratios (HR) are for a change between the 25th and 75th percentile of the continuous scores. Changes in likelihood ratio values (LR*χ*^2^) were used to measure and compare the relative amount of information of one score compared to the other. *P* values were two-sided, based on normal approximation, and all confidence intervals were at the 95 % level. Analyses were performed using STATA version 13.1 (College Station, Texas, USA).

## Results

940 postmenopausal women with hormone receptor-positive primary breast cancer were included in this analysis. Baseline demographics are presented in Table 1. Median age was 63.6 years (IQR 57.9–70.7) and median BMI was 26.6 kg/m^2^ (IQR 23.5–29.9). For this analysis, we used age tertiles and conventional BMI groups to determine the impact of prognostic performance of the scores in each group. All other baseline factors were used as dichotomous variables in our analyses and are shown in Table 1.

**Table 1:** Baseline demographics and number of distant recurrence

	Number of women (*N* = 940)	Number of distant recurrence (%)
Age (years), median (IQR)	63.6 (57.9−70.7)	
1st tertile (*N* = 314), median (IQR)	55.7 (53.1−57.9)	33 (10.5)
2nd tertile (*N* = 313), median (IQR)	63.6 (61.6−65.7)	51 (16.3)
3rd tertile (*N* = 314), median (IQR)	73.5 (70.7−76.8)	70 (22.4)
BMI (kg/m^2^), median (IQR)	26.6 (23.5−29.9)	
≤25 (*N* = 314), median (IQR)	22.5 (21.2−23.8)	49 (15.6)
25–30 (*N* = 339), median (IQR)	27.4 (26.1−28.6)	59 (17.4)
>30 (*N* = 212), median (IQR)	32.8 (31.2−34.9)	34 (16.0)
Prior HRT (%)	340 (36.2 %)	41 (12.1)
Never smokers (%)	477 (50.7 %)	79 (16.6)
Hysterectomy (%)	208 (22.1 %)	33 (15.9)
Radiotherapy (%)	639 (68.0 %)	103 (16.1)
Mastectomy (%)	390 (41.5 %)	94 (24.1)

### Age

**Fig. 1:**
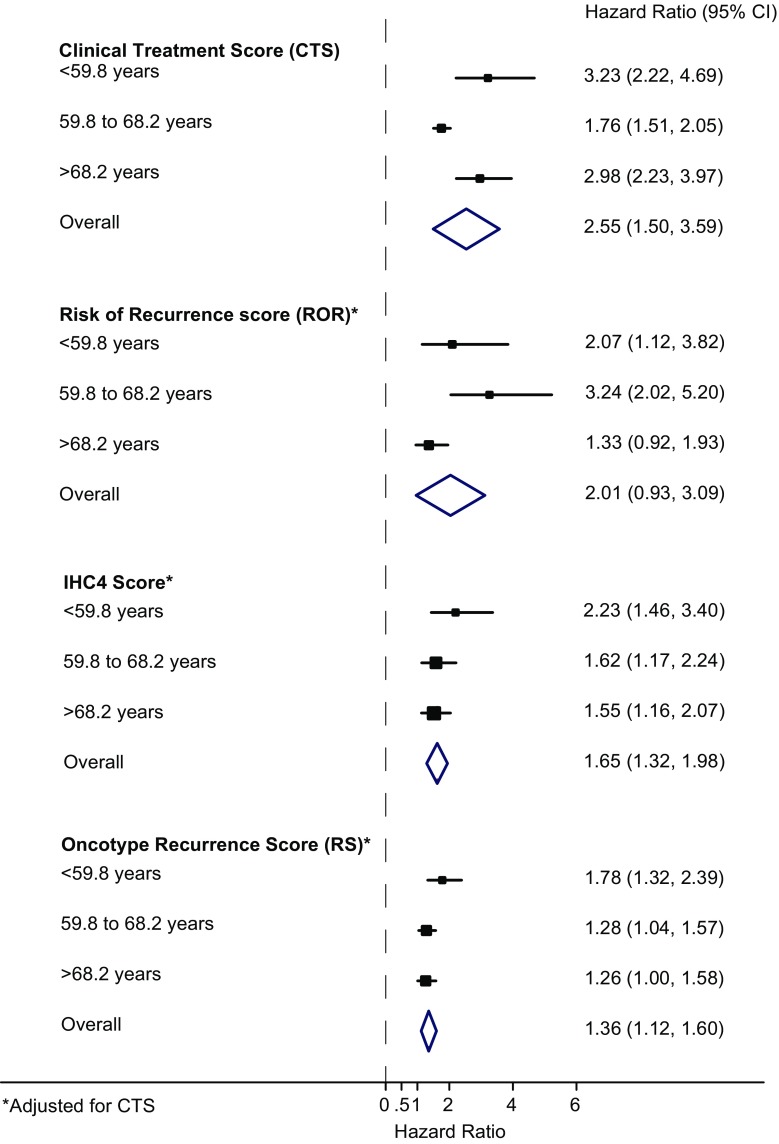
Forest plot for prediction of distant recurrence according to signature and age groups

Overall, age as a continuous variable was a significant risk factor for distant recurrence in all patients (for a change in one standard deviation (SD): HR = 1.73 (1.36–2.20), *P* < 0.001), and those with node-negative/HER2-negative disease (for a change in one SD: HR = 1.92 (1.28–2.89), *P* = 0.002). Table 2 shows the prognostic performance of each score according to age tertile at baseline. In the univariate analysis, the largest effect sizes for the CTS were seen in the youngest [HR = 3.23 (2.22–4.69)] and the oldest age group [HR = 2.98 (2.23–3.97)], whereas the CTS was less prognostic for women between the ages of 59.8 and 68.2 years (Fig. 1). However, an interaction test for age and CTS was statistically not significant (*P*_interaction_ = 0.056). For the ROR, age added most prognostic information for women in the 2nd tertile [HR = 4.51 (2.87–7.10)], and the score was less predictive for distant recurrence in the other two age groups in the univariate analysis (*P*_interaction_ = 0.055) (Table 2). A different picture was seen for the IHC4 and Oncotype RS, where the largest effect sizes and the most prognostic value were observed in the youngest age group (HR = 3.01 (1.99–4.53) and HR = 2.16 (1.62–2.87), respectively). Both scores were significantly less prognostic for distant recurrence in women aged 59.8 years or older (Table 2). A significant interaction for age and IHC4 was observed (*P*_interaction_ = 0.033), but not so for age and RS (*P*_interaction_ = 0.056). For the bivariate analyses, each score was added to the CTS to see what additional prognostic information was provided by each score in each age group. Results are shown in Table 2 and graphically in Fig. 1. The ROR score added significant prognostic information when adjusted for the CTS for women between the ages of 59.8 and 68.2 years [HR = 3.24 (2.02–5.20)], but was less predictive in the youngest age group and did not add any significant prognostic information for women older than 68.2 years of age, HR = 1.33 (0.92–1.93) (Table 2; Fig. 1). For the IHC4 and Oncotype RS, similar results were seen as in the univariate analysis. In the bivariate analysis, both scores showed the largest effect size in the youngest age group (Table 2), with significant decreasing prognostic performance with increasing age (*P*_trend for both_ ≤ 0.0001) (Fig. 1).

**Table 2:** Hazard ratios (HRs) and likelihood ratio tests (LR*χ* ^2^) for all four scores according to age tertiles and BMI group for the univariate and bivariate analyses

Univariate analysis	CTS		ROR		IHC4		RS	
Age (years) (tertiles)	HR (95 % CI)	LR*χ* ^2^	HR (95 % CI)	LR*χ* ^2^	HR (95 % CI)	LR*χ* ^2^	HR (95 % CI)	LR*χ* ^2^
≤59.8 (*N* = 314)	3.23 (2.22−4.69)	34.24	3.87 (2.21−6.78)	23.21	3.01 (1.99−4.53)	25.08	2.16 (1.62−2.87)	22.55
59.8–68.2 (*N* = 313)	1.76 (1.51−2.05)	41.23	4.51 (2.87−7.10)	44.74	1.67 (1.23−2.26)	10.00	1.39 (1.16−1.66)	9.64
>68.2 (*N* = 313)	2.98 (2.23−3.97)	50.17	1.83 (1.28−2.60)	11.39	1.64 (1.25−2.15)	12.05	1.38 (1.11−1.73)	7.20
Bivariate analysis (in addition to CTS)								
Age (years) (tertiles)				∆LR*χ* ^2^		∆LR*χ* ^2^		∆LR*χ* ^2^
≤59.8 (*N* = 314)			2.07 (1.12−3.82)	5.50	2.23 (1.46−3.40)	13.63	1.78 (1.32−2.39)	13.32
59.8–68.2 (*N* = 313)			3.24 (2.02−5.20)	24.95	1.62 (1.17−2.24)	7.61	1.28 (1.04−1.57)	4.69
>68.2 (*N* = 313)			1.33 (0.92−1.93)	2.28	1.55 (1.16−2.07)	8.11	1.26 (1.00−1.58)	3.62
Univariate analysis BMI (kg/m^2^) (tertiles)								
≤25 (*N* = 314)	2.54 (1.97−3.30)	42.32	3.01 (1.88−4.84)	21.54	2.37 (1.70−3.31)	23.76	1.74 (1.35−2.25)	15.47
25–30 (*N* = 339)	2.10 (1.67−2.60)	38.89	3.21 (2.21−4.67)	38.16	1.72 (1.31−2.26)	13.88	1.49 (1.26−1.76)	15.48
>30 (*N* = 212)	2.64 (2.02−3.45)	44.43	4.23 (2.31−7.74)	24.5	1.65 (1.11−2.46)	5.57	1.18 (0.85−1.64)	0.87
Bivariate analysis (in addition to CTS)								
BMI (kg/m^2^) (tertiles)				∆LR*χ* ^2^		∆LR*χ* ^2^		∆LR*χ* ^2^
≤25 (*N* = 314)			1.92 (1.19−3.09)	7.29	2.02 (1.43−2.84)	15.09	1.54 (1.18−2.02)	9.02
25–30 (*N* = 339)			2.33 (1.55−3.51)	16.62	1.66 (1.24−2.22)	10.75	1.43 (1.18−1.72)	10.77
>30 (*N* = 212)			2.40 (1.21−4.74)	6.63	1.43 (0.92−2.23)	2.34	1.07 (0.77−1.48)	0.15

### BMI

**Fig. 2:**
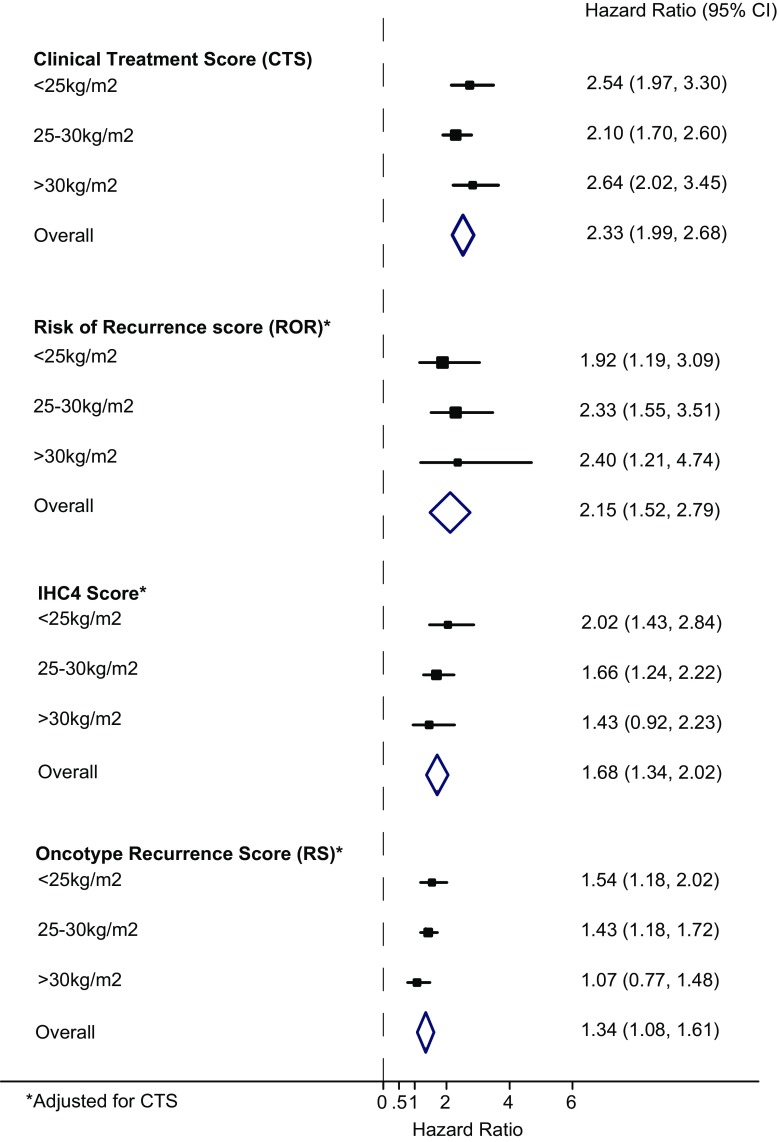
Forest plot for prediction of distant recurrence according to signature and BMI groups

BMI as a continuous variable was not a significant risk factor for distant recurrence (for a change in one SD: HR = 1.12 (0.90–1.38), *P* = 0.3). In the univariate analysis, CTS provided similar amount of prognostic information across all three BMI groups (Table 2). For the ROR score, the most prognostic information for distant recurrence in the univariate analysis was added for women with a BMI between 25 and 30 kg/m^2^. Different results were observed for the IHC4 and Oncotype RS, where the largest effect sizes were seen in the lowest BMI tertile (HR = 2.37 (1.70–3.31), HR = 1.74 (1.35–2.25), respectively), and decreasing prognostic information was added with increasing BMI, although a trend test across BMI groups was not significant (Table 2). No significant interaction was observed for any score with BMI (all *P*_interaction_ > 0.05). In the bivariate analysis, the most prognostic value for distant recurrence by all three scores was found in women with a BMI between 25 and 30 kg/m^2^, and all scores were significantly less predictive in women with a BMI over 30 kg/m^2^ (Table 2; Fig. 2). To account for the inclusion of age in the CTS, we performed all analyses without adjusting the multi-gene signatures for the CTS and observed very similar results (Table 2).

The risk factor analysis according to HRT use, radiotherapy, smoking status, hysterectomy, or mastectomy did not reveal any differences in the prognostic performance of the scores (data not shown).

## Discussion

Many multi-gene signatures have been developed for the prediction of (distant) recurrence in women with early hormone receptor-positive breast cancer. They all have shown to add significant prognostic information for recurrence in different clinical settings [2, 4, 8]. These scores have been developed for a variety of different clinicopathological groups, e.g. women with ER-positive breast cancer or for those with node-negative disease. However, no other non-clinical factors have been taken into account when assessing the prognostic value of these multi-gene scores.

Our results show that age is an important non-clinical factor when assessing the prognostic performance of clinical, immunohistochemical, and multi-gene scores. Age was a significant risk factor for distant recurrence. Similar results were reported by the TEAM trialist group [23], which reported an increased risk of distant recurrence in elderly patients with hormone receptor-positive breast cancer treated with endocrine therapy alone. In the transATAC trial, elderly women were less adherent to their treatment allocation compared with younger women, which may partly explain the higher risk of distant recurrence. However, older women also had higher oestrogen and Ki67 levels than younger women, which could also contribute to an increase risk of recurrence. Increased levels of oestrogen and Ki67 in older women were also observed by Paik et al. [2], but in contrast they found that older women had fewer recurrences than younger women.

In our study, the prognostic performance of all scores was lowest for the older patients in the univariate and bivariate analyses. This decrease in performance with age was especially pronounced for the IHC4 and the *Oncotype* Dx RS, for which the most prognostic information was added in women aged 59.8 years or younger, with a statistically significant trend observed with increasing age. This trend toward decreased performance of multi-gene signatures with increasing age may be explained in part by a difference in tumour biology of older patients. In elderly women, immunosenescence plays an important role [24, 25] in tumourigenesis and progression. This altered immune state of older women appears to lead to altered tumour biology and may also result in worse performance of multi-gene signatures than in younger women. In addition, as was mentioned above, significantly higher levels of oestrogen and Ki67 were observed in older women, and these older women were also more likely to have poorly differentiated tumours than younger women. Both the IHC4 and Oncotype RS incorporate information on Ki67 and ER levels, and the observed increase in these levels in older women might explain the diminishing effect of these scores with increasing age. Our results suggest that multi-gene signatures may not accurately capture the risk of recurrence in older women, but further validation to confirm this finding is needed.

A similar picture, but less pronounced, was observed for BMI. The IHC4 and Oncotype RS were most prognostic in leaner women (BMI <25 kg/m^2^), whereas the Prosigna ROR score added most value in women with a BMI between 25 and 30 kg/m^2^. A recent study by Creighton et al. investigated the impact of obesity on the expression profiles of 662 tumours and found that obesity was correlated with patterns of gene expression, specifically gene signatures for insulin-like growth factor (IGF) signalling and to a lesser extent lower levels of ER [26]. In our study, obese women also had lower levels of ER, although the difference compared with leaner women was not statistically significant. It is possible that the changes in gene expression associated with obesity may be related to the decreased prognostic performance of immunohistochemical and multi-gene scores. Taking this finding together with the fact that obese women with early-stage breast cancer have a poorer prognosis [27], the use of these multi-gene signatures in this patient group should be investigated further.

Strengths of this analysis include the large sample size, long-term follow-up for all patients, and the availability of all scores for all patients. We performed the comparison of standardised prognostic assays routinely used in the clinic in a well-characterised set of samples. Limitations included that all women are from the United Kingdom, and therefore our results may not be translated for populations in other countries. All demographics were only collected at baseline, and we do not have any data on change of BMI with follow-up time. Furthermore, we have to acknowledge that this analysis was of a retrospective nature. Lastly, in a number of cases, multiple comparisons were made and caution is needed in interpreting those results. However, in the univariate analysis, the majority of tests and comparisons were highly statistically significant at the 1 % level, even after correction for multiple comparisons (nominal *P* < 0.001). For subgroup analyses, heterogeneity tests are more important [28], and no heterogeneity was observed between subgroups.

In summary, our results from this retrospective analysis show that factors other than tumour biology and clinical characteristics are important when assessing recurrence risk by multi-gene signatures in women with hormone receptor-positive breast cancer. Patient’s biologic characteristics need to be taken into account as well for an adequate risk categorisation by these scores. Further validation of our results is needed before they can be implemented in clinical decision making.
